# Routine examination of gallbladder specimens after cholecystectomy: a single-centre analysis of the incidence, clinical and histopathological aspects of incidental gallbladder carcinoma

**DOI:** 10.1007/s12672-021-00399-5

**Published:** 2021-02-15

**Authors:** Davide Di Mauro, Amira Orabi, Aye Myintmo, Alex Reece-Smith, Shahjehan Wajed, Antonio Manzelli

**Affiliations:** Department of Upper GI Surgery, Royal Devon and Exeter NHS Foundation Trust, Barrack Road, Exeter, EX25DW UK

**Keywords:** Gallbladder cancer, Analysis, Histopathological examination, Survival

## Abstract

**Background:**

Gallbladder carcinoma is often found incidentally on histopathologic examination after cholecystectomy—this is referred as incidental gallbladder carcinoma (IGC). Routine vs selective histopathological assessment of gallbladders is under debate and this study evaluates the role of regular specimens’ examination, based on a single-centre analysis of incidence, clinical and histopathological aspects of IGC.

**Methods:**

Patients who underwent cholecystectomy, between July 2010 and January 2020, were considered. Exclusion criteria were age under 18 and preoperative diagnosis of GB carcinoma. Demographic, clinical and histopathological data were retrospectively collected, continuous variables with a normal distribution were evaluated with Student’s t-test and ANOVA.

**Results:**

Some 5779 patients were included. The female/male ratio was 2.5:1. Chronic cholecystitis (CC) was the most common finding on specimens (99.3%), IGC was found in six cases (0.1%). In the latter group, there were 5 women and patients were older than those with benign disease—73.7 $\pm$ 5.38 years vs 55.8 $\pm$ 0.79 years (p < 0.05). In all the cases, the GB was abnormal on intraoperative inspection and beside cancer, histopathology showed associated CC and/or dysplasia. Upon diagnosis, disease was at advanced stage—one stage II, one stage IIIA, one stage IIIB, three stage IVA. Two patients are alive, three died of disease progression—median survival was 7 months (range 2–14).

**Conclusions:**

In this series, ICG was rare, occurred most commonly in old adult women and was diagnosed at an advanced stage. In all the cases, the GB was abnormal intraoperatively, therefore macroscopic GB anomalies demand histopathological assessment of the specimen.

## Introduction

Gallbladder (GB) carcinoma represents the most common cancer of the biliary tract and accounts for the fifth cause of gastro-intestinal cancer [1]. Its geographical distribution is not homogenous, as its prevalence is higher in Japan, in some regions of India, South America and Eastern Europe, while it is relatively rare in Northern Europe and America [1, 2]. The disease represents a diagnostic and clinical challenge, since its presentation is often non-specific [3] and in two third of the cases, it is found incidentally on histopathologic examination after cholecystectomy [4]—in which case it is known as *incidental gallbladder carcinoma* (IGC). Prognosis is poor, as the overall 5-year survival rate is less than 5% [4, 5].

The incidence of IGC in GB specimens ranges between 0.2 and 3.3% [6] and histopathological examination of cancerous GB is necessary to stage the disease, thus allowing for the most appropriate treatment. Given the low disease incidence, there is an argument as to whether routine histological assessment of all the specimens, after cholecystectomy, is necessary.

This study aims to evaluate the role of regular histopathological examination of the GB, based on a single-centre analysis of the incidence, clinical and histopathological aspects of IGC.

## Methods

This was a single-centre observational study, local board approval was obtained. Data from all the patients who underwent cholecystectomy at the Royal Devon and Exeter NHS Foundation Trust, United Kingdom, between July 2010 and January 2020, were retrospectively evaluated. Exclusion criteria were preoperative suspicion of GB cancer and patient’s age under 18. Demographics, indications of surgery, intraoperative findings, histopathology results, and clinical outcomes of IGC, were analyzed; GB carcinoma was staged according to the American Joint Committee on Cancer, 8th edition [7].

**Fig. 1:**
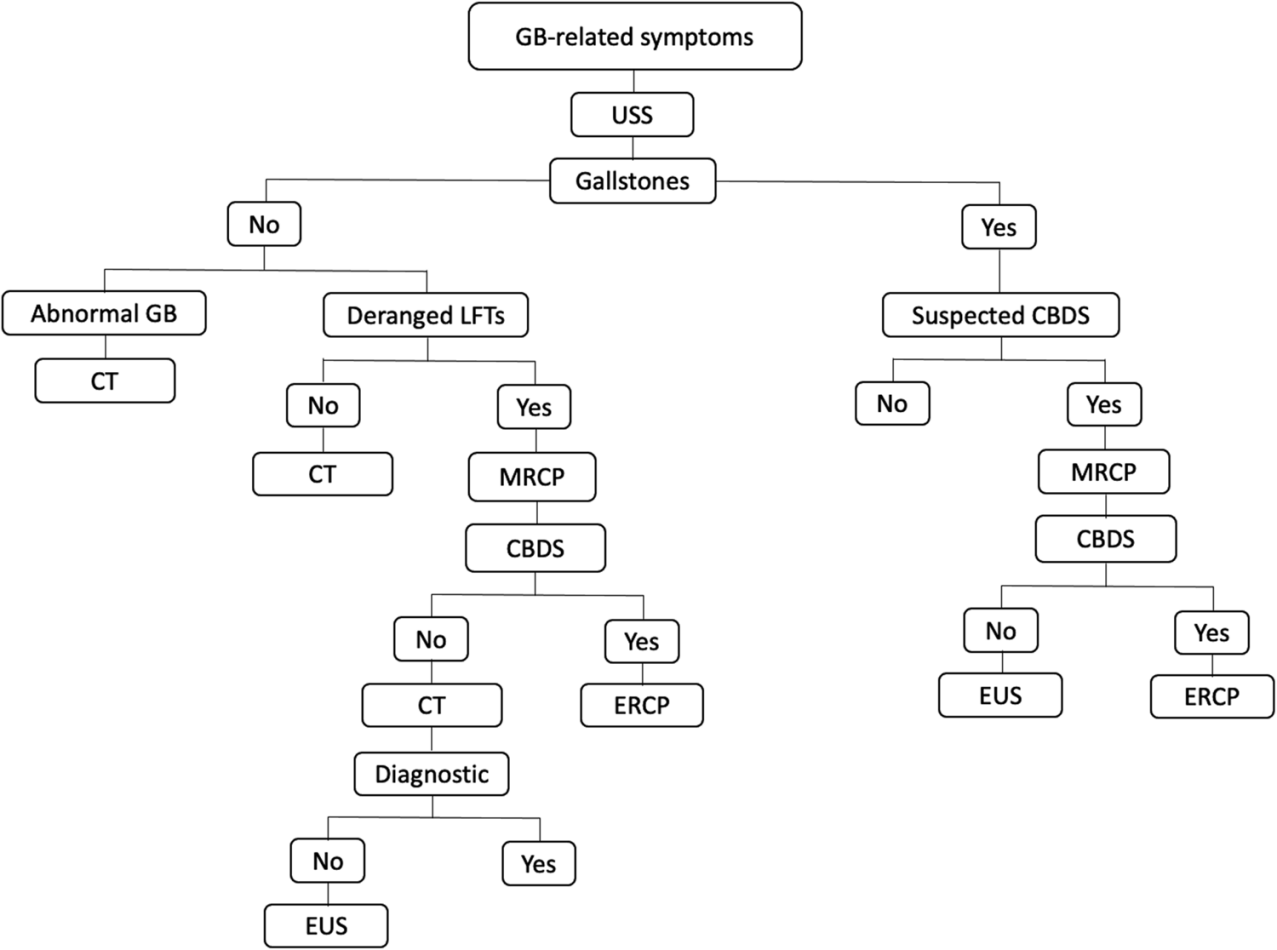
Diagnostic work-up in the elective setting. *GB* gallbladder, *USS* ultrasound scan of the abdomen, *LFTs* liver function tests, *CBDS* common bile duct stones, *CT* computed tomography scan of the abdomen, *MRCP* magnetic resonance cholangio-pancreatography, *ERCP* endoscopic retrograde cholangio-pancreatography, *EUS* endoscopic ultrasound

**Fig. 2:**
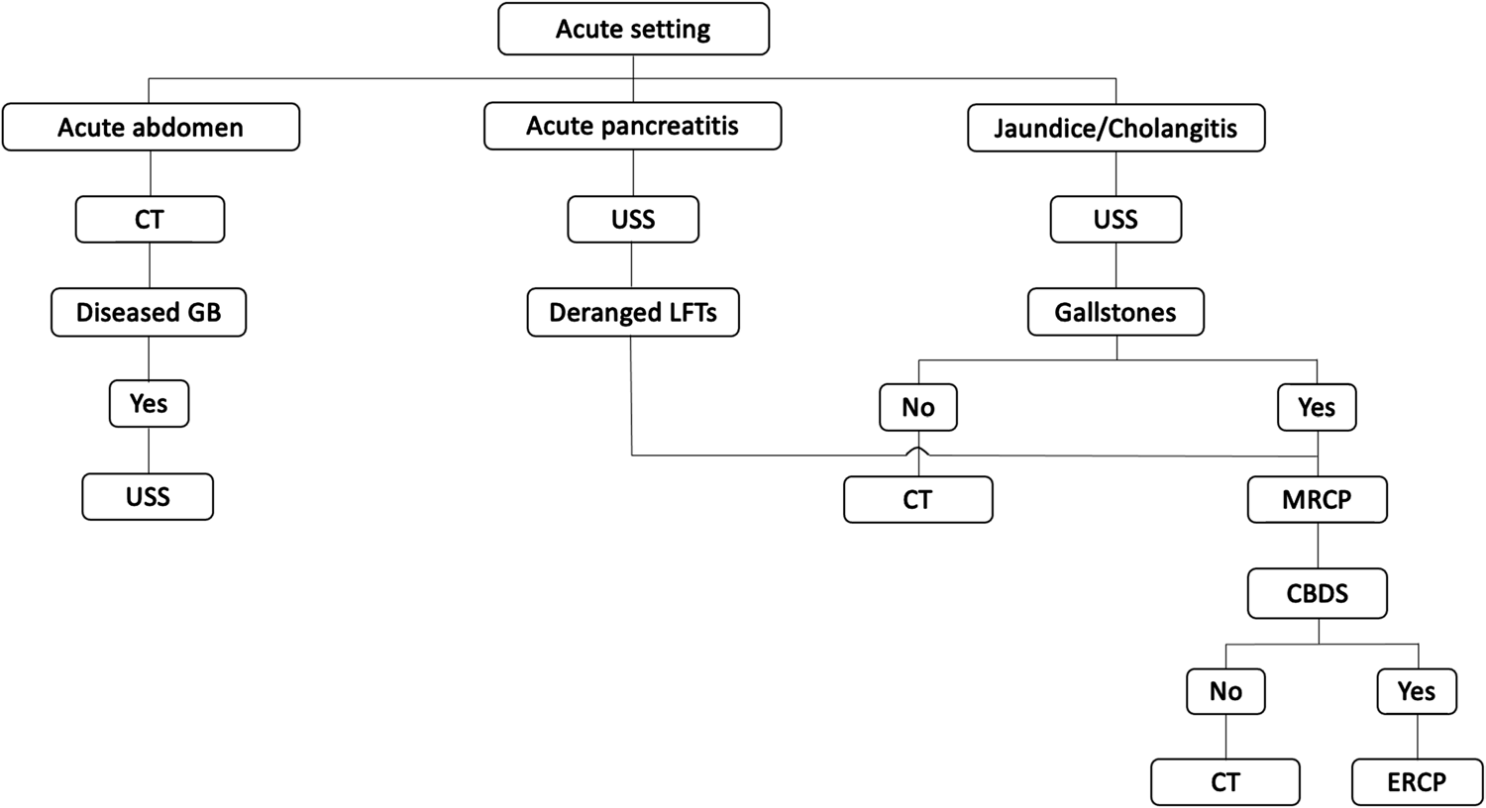
Diagnostic work-up in the acute setting. *CT* computed tomography scan of the abdomen, *GB* gallbladder, *USS* ultrasound scan of the abdomen, *LFTs* liver function tests, *MRCP* magnetic resonance cholangio-pancreatography, *CBDS* common bile duct stones, *ERCP* endoscopic retrograde cholangio-pancreatography

At our Institution, all the patients undergoing cholecystectomy are assessed with preoperative ultrasound scan of the abdomen; computed tomography (CT) scan of the abdomen, magnetic resonance cholangiopancreatography (MRCP) and/or endoscopic ultrasound scan, are performed when the GB morphology is abnormal, to rule out the presence of common bile duct stones or in case of diagnostic uncertainty (Figs. 1 and 2). In most of the cases, surgery is performed laparoscopically, through a standard 4 trocars technique and the GB specimen is sent for histopathological examination on a routine basis.

For statistical analysis, continuous variables with a normal distribution were described using the mean ± standard error; means between groups were computed with the Student’s T-test (two groups) and ANOVA (more than two groups). Data that were not normally distributed were described as median and interquartile range (IQR); categorical data were given as absolute numbers and percentages. Significance was set when p < 0.05 (SPSS Statistics version 20, IBM, Armonk, NY).

## Results

Overall, 5802 patients who underwent cholecystectomy were identified; 23 were excluded because of preoperative suspicion of GB cancer (4) and age under 18 (19), respectively; therefore, 5779 were considered for the study.

### Overall patients’ demographics

There were 4161 females, 1618 males and the male/female ratio was 2.5:1; mean age was 54.6 ± 0.2 years. Main indications for surgery were symptomatic gallstones (99.3%) and GB polyps. On histopathological examination, abnormalities were found in 99.7% of patients (Table 1); chronic cholecystitis (CC) was the most common finding (97.4%), followed by xanthomatous cholecystitis (1.2%) and dysplasia (0.6%), while IGC was found in 6 cases (0.1%). One patient was diagnosed with non-Hodgkin lymphoma.

**Table 1:** Demographic and histopathological data

Histopathology	Number (%)	Gender	Mean age ± SE^b^	p-value
Chronic cholecystitis	5628 (97.4)	F 4053 M 1575	54.7 ± 0.22	NS^c^
Xanthomatous cholecystitis	71 (1.2)	F 38 M 33	56.6 ± 1.7	NS^c^
Dysplasia	38 (0.6)			
Low grade	36	F 31	55.3 ± 2.7	NS^c^
High grade	2	M 7		
Normal	18 (0.31)	F 14 M 4	45.9 ± 2.95	NS^c^
Acute cholecystitis	16 (0.27)	F 9 M 7	68.4 ± 2.92	NS^c^
Carcinoma (IGC^a^)	6 (0.1)	F 5 M 1	73.7 ± 5.38	< *0.05*^d^
Hyalinizing cholecystitis	1	F	56	–
Lymphoma	1	F	63	–

^a^Incidental gallbladder cancer

^b^Standard error

^c^Not significant

^d^Analysis of variance (ANOVA) test

#### IGC

There were five women and patients in this group were older than those with benign disease—73.7 $\pm$ 5.38 years vs 55.8 $\pm$ 0.79 years (p < 0.05) (Table 1). In all cases, indication of surgery was gallstone disease (Table 2) and median duration of abdominal symptoms was 7 months (range 2–17, IQR 9.5). Preoperative abdominal CT scan and MRCP were performed in the acute setting in three patients—cholangitis (2), pancreatitis (1), respectively; in the remaining 3, CT was done to rule out causes of abdominal pain other than gallstones, while 2 had also MRCP because of mild transient elevation of the serum liver function tests. Overall, three patients had abnormal GB on preoperative imaging—thick wall (2) and adenomyomatosis (1), respectively. In all the cases, cholecystectomy was performed laparoscopically. During surgery, a GB mass was identified in one patient, in the other 5 the organ was thick-walled, with local dense adhesions.

**Table 2:** Characteristics of patients with incidental gallbladder cancer

Patient	Gender	Age	Indication of surgery	GB^a^ on preoperative imaging	Histopathology	AJCC^b^ stage	Treatment	Alive	Survival (months)
1	M	53	Biliary colic	Thick-walled	CC	II	Surgery	Y	17
2	F	80	Pancreatitis	Thick-walled	CC	IVA	Chemotherapy	N	2
3	F	80	Cholangitis	Normal	CC + dysplasia	IVA		N	7
4	F	72	Biliary colic	Normal	CC	IIIB	Chemotherapy	N	7
5	F	66	Cholangitis	Normal	Dysplasia	IVA	Chemotherapy	N	14
6	F	91	Biliary colic	Adenomyomatosis	CC + dysplasia	IIIA		Y	23

*CC* Chronic cholecystitis

^a^Gallbladder

^b^American Joint Committee on Cancer

Histopathological examination of the GB demonstrated other abnormalities in association with cancer, in all the patients—CC (3), dysplasia (1), CC and dysplasia (2).

The AJCC staging was as follows: stage II (1), stage IIIA (1), stage IIIB (1), stage IVA (3). The patient with stage II disease had underwent excision of liver segments IVb-V and lymphadenectomy of the liver pedicle. Three patients—one stage IIIB, two stage IVA—had palliative chemotherapy (Table 2).

Median follow-up was 15 months (range 5–23, IQR 16.5). Two patients—stage II and IIIA, respectively—are alive and disease-free at the time of writing, the remaining died of disease progression and their median survival was 7 months (range 2–14, IQR 9).

## Discussion

Outcome of GB cancer depends on disease stage, as the tumour extent into the GB wall correlates to the risk of peritoneal metastases [8]; therefore, it is crucial to diagnose the disease in the early stage. The 1-year survival is reported as 100% for T1, 75% for T2, 40% for T3, 0% for T4, respectively [9].

In our series, the incidence of IGC was 0.1%, which is lower than that reported in the literature [6]. Perhaps, the fact that the study was conducted in a low incidence geographical area [10], may explain that. Moreover, beside CC that was the most common finding, histopathological examination found xanthomatous cholecystitis and dysplasia in 109 patients; in line with other published series [11, 12], the authors argue that a high volume of cholecystectomies might prevent GB cancer by interrupting the progression of chronic inflammation and dysplasia towards malignant transformation, thus leading to a reduced incidence. The study was not powered for such evaluation and further research is needed to corroborate or confute such a hypothesis.

IGC occurred more often in women who were older than those with benign conditions; such a figure is in line with other series [13, 14], although in high prevalence areas, the disease seems to occur at a younger age [12]. Gallstone diseases were the main indication of surgery and the median duration of abdominal symptoms was 7 months. The association of gallstones and GB cancers is well described, although it is still unclear whether gallstones are a risk factor or may somewhat facilitate the occurrence of the disease [14, 15]. In all six patients, the GB looked abnormal during surgery; similar results were observed in other studies [16]. Beside cancer, histopathological examination showed associated CC and/or dysplasia; whilst CC was the most common finding, dysplasia was found in isolation in one patient and associated with CC in 3. This aspect seems to corroborate the well described link between these alterations and GB cancer, in fact it is suggested that the chronic inflammation of the GB mucosa either stimulates or facilitates its transformation into dysplastic epithelium first, then cancer [6, 16–21].

Upon diagnosis, all the patients had advanced disease; in some published series, stage I and stage II disease occurred in more than 60% of patients [8, 9], whereas other authors reported more advanced stages [6, 22]. An argument to explain such a result is that gallstone-related symptoms and non-specific preoperative imaging results, may contribute to a relative delayed diagnosis of cancer. This aspect had been acknowledged in the published literature [14, 23].

Given the relative low incidence of IGC, there is ongoing debate as to whether assessment of all the GB specimens should be done on a regular basis. Arguments in favour of a routine approach include the lack of potential oversight of cases [2, 24], accurate disease staging [25] and medico-legal implications in case of disputes or diagnostic uncertainty [26]. A more selective approach entails the examination of the specimen only if the GB looks abnormal pre- or intraoperatively (i.e., in the presence of a thick, fibrosed wall, local dense adhesions). Advocates of such a strategy claim that IGC is unlikely to be found in a normal-looking GB [16, 27]. Also, since early-stage disease is the most common finding of IGC, cholecystectomy alone would be curative and no further treatment would be needed [28, 29]. Finally, this approach would reduce time and costs for the assessment of the specimens.

In conclusion, in our series the incidence of IGC is low and disease occurs more often in female patients who are older than those with benign gallbladder diseases. Symptoms are related to gallstones and the GB is abnormal on both pre- and intraoperative assessment. Accurate staging is necessary to provide the most appropriate treatment and despite views on routine histopathological examination are still under debate, macroscopic GB abnormalities demand assessment of the specimen.
